# Understanding Host-Adherent-Invasive *Escherichia coli* Interaction in Crohn's Disease: Opening Up New Therapeutic Strategies

**DOI:** 10.1155/2014/567929

**Published:** 2014-12-15

**Authors:** Allison Agus, Sébastien Massier, Arlette Darfeuille-Michaud, Elisabeth Billard, Nicolas Barnich

**Affiliations:** ^1^Clermont Université, M2iSH, UMR 1071 INSERM/Université d'Auvergne, CBRV, 28 place Henri Dunant, 63001 Clermont-Ferrand, France; ^2^Unité Sous Contrat 2018, Institut National de la Recherche Agronomique, 63001 Clermont-Ferrand, France; ^3^Institut Universitaire de Technologie, Génie Biologique, 63172 Aubière, France

## Abstract

A trillion of microorganisms colonize the mammalian intestine. Most of them have coevolved with the host in a symbiotic relationship and some of them have developed strategies to promote their replication in the presence of competing microbiota. Recent evidence suggests that perturbation of the microbial community favors the emergence of opportunistic pathogens, in particular adherent-invasive *Escherichia coli* (AIEC) that can increase incidence and severity of gut inflammation in the context of Crohn's disease (CD). This review will report the importance of AIEC as triggers of intestinal inflammation, focusing on their impact on epithelial barrier function and stimulation of mucosal inflammation. Beyond manipulation of immune response, restoration of gut microbiota as a new treatment option for CD patients will be discussed.

## 1. Introduction

A population of 10^14^ commensal microorganisms composes the human gut microbiota. Their genome (also named metagenome or microbiome) represents, in terms of gene number, 150-fold the human genome [1]. Microbiota influences physiology and metabolism within the body. In addition to influencing the metabolism of the host, microbiota could also be involved in various pathological mechanisms. Both development and activation of our mucosal immune system in GI tract depend on this complex consortium of microorganisms [2]. Recent evidence has pointed to the role of gut microbiota in various human diseases such as IBD, colon cancer, type 1 diabetes, insulin resistance, nonalcoholic fatty-liver disorders, asthma, and allergies. Thus, it is important to understand the involvement of microbiota in the etiology of such diseases by characterizing species that compose a “healthy” microbiota [3–8].

In inflammatory bowel diseases (IBD), including Crohn's disease (CD) and ulcerative colitis (UC), a dysfunction of the immune response to gut microbiota occurs in a context of host genetic predisposition. CD is a chronic and commonly disabling inflammatory disorder of the intestine whose prevalence and incidence increase in the developed countries [9]. IBD preferentially occurs in the colon and the distal ileum, intestinal portions harboring the largest concentration of microorganisms. Involvement of microbiota in IBD pathogenesis was supported by experiments performed in germ-free animal models since the presence of microbiota was required to trigger intestinal inflammation in various models (IL-10 and IL-12 knock-out mice, chemically DSS- and TNBS-induced colitis) [10, 11]. More recently, genetic evidence has shown associations between IBD and genes involved in antibacterial response, such as NOD2, autophagy-related genes, and the IL23R pathway involved in Th17 polarization. Several nonexclusive mechanisms could drive the pathogenic immunologic response to microbiota: (i) involvement of microbial pathogens that induce intestinal inflammation, such as traditional pathogens (*Mycobacterium avium* subspecies* paratuberculosis*) or functional alteration of commensal bacteria (adherent-invasive* Escherichia coli*, toxigenic* Bacteroides fragilis*, etc.), (ii) dysbiosis of commensal microbiota, with a depletion of protective bacterial species versus an enrichment of harmful species, (iii) host genetic inability to contain commensal microbiota due to defective intracellular bacterial killing and impaired intestinal barrier function, and (iv) defective host immunoregulation.

## 2. Importance of* Escherichia coli* as Triggers of Intestinal Inflammation in Crohn's Disease

An altered gut microbiota has long been suspected to play an important part in the pathogenesis of IBD. The evidence that enteric bacterial antigens continuously drive chronic, immune-mediated colitis and ileitis is provided by rodent models of spontaneous or induced intestinal inflammation [12].

### 2.1. Dysbiosis

A general dysbiosis of gut microbiota has been well established in IBD patients by both culture-dependent and culture-independent techniques [13, 14]. This altered composition of the commensal bacterial populations may result from a modulation of oxygen levels in inflamed gastrointestinal tract, leading to an overgrowth of bacteria having proinflammatory properties and/or to a decrease of beneficial commensal species [15]. Although a specific pattern of dysbiosis in IBD patients is difficult to establish, many studies have reported an increase in the abundance of Proteobacteria and Bacteroidetes and a decrease in Firmicutes [16]. In samples from multiple gastrointestinal locations in a large pediatric CD cohort collected prior to treatment in new-onset cases, an increased representation of Enterobacteriaceae, Veillonellaceae, Fusobacteriaceae, and Pasteurellaceae populations and a reciprocal decrease in Bacteroidales, Clostridiales, and Erysipelotrichales were strongly associated with disease status [17]. This study also indicated that, at the early stage of the disease, analysis of the rectal mucosal-associated microbiota could help to diagnose CD. More recently, analysis of fungal microbiota showed that its composition differs in inflamed and noninflamed area, suggesting that gut fungal exploration could be used to evaluate CD disease activity [18]. Now, intestinal microbiota should be investigated at the ecological level. A recent study reported that, on intestinal mucosal surface, bacterial community is organized into five highly conserved modules in human, two of them displaying distinct metabolic functionalities and being reciprocally associated with IBD. An integrative view of microbial ecology associated with IBD status of individual patients during disease was possible based on the analysis of microbial modules organization [19].* Bacteroides fragilis,* a human symbiont, had anti-inflammatory effects,* via* expression of polysaccharide A (PSA) in* Helicobacter hepaticus*-induced colitis in mice [2, 4]. On resected ileal Crohn's mucosa, decreased levels of* Faecalibacterium prausnitzii* population were associated with endoscopic postoperative recurrence [20].* F. prausnitzii,* a beneficial bacteria, is known to induce an immunoregulatory cytokine secretion in peripheral blood mononuclear cells with high amounts of IL-10 and low amounts of IL-12 [20, 21]. In the fecal microbiota of UC patients, decreased levels of the butyrate-producing* Roseburia hominis* and* Faecalibacterium prausnitzii* were recently reported [22]. Distinct ratio of* F. prausnitzii* and* E. coli* has been reported in ileal and colonic CD, respectively, therefore allowing to consider this ratio as a promising biomarker for differential diagnosis and personalized treatment [23].

### 2.2. Traditional Pathogens

Molecular techniques have identified specific pathogenic agents playing a role in inflammation of IBD. Much research has shown a higher prevalence of* Mycobacterium avium paratuberculosis*,* Helicobacter *species, and* Campylobacter concisus* in IBD patients than in control subjects [24–26]. Other bacterial pathogens are also suspected of involvement in these diseases, such as* Fusobacterium*,* Klebsiella*,* Salmonella,* and* Yersinia *[27–31]. Despite recurrent indications that traditional pathogens could be involved in IBD, their role as causative agents of CD remains very uncertain. However, it is possible that only a subset of patients is concerned and investigation is needed especially in those with defects in intracellular killing of bacteria due to genetic polymorphisms (ATG16L1, IGRM, or NCF4).

### 2.3. Adherent-Invasive* E. coli*

Over the last 10–15 years, the microbe that has attracted the most attention, with respect to CD etiology, is* Escherichia coli *[32, 33]. Overgrowth of* E. coli *population in inflammatory bowel disease patients is currently unexplained but may be related to increased production of reactive nitrogen species allowing nitrate respiration, which confers* E. coli* a fitness advantage [34]. A specific pathogenic group of* E. coli*, called adherent-invasive* E. coli* (AIEC), has been extensively implicated in human CD and is currently one of the most exciting players in the pathogen story (Table 1) [23, 35–66]. AIEC bacteria strongly adhere to and invade intestinal epithelial cells (IEC) by a mechanism involving microtubule polymerisation and actin recruitment [67], inducing inflammatory cytokine secretion [68]. AIEC survive and replicate inside macrophages, induce an important secretion of TNF-*α*, and promote granuloma formation* in vitro *[69–71]. AIEC strains induce IL-1*β* *via *an NLRP3-dependent mechanism, but their elimination by macrophages is independent of NLRP3 [72]. Invasiveness of intracellular* E. coli* strains into the intestinal mucosa and IL-1*β* production may contribute to CD and UC pathogenesis. AIEC strains have been shown to be the cause of granulomatous colitis in Boxer dogs and to induce granulomas, similar to early epithelioid granulomas,* in vitro* [71, 73]. AIEC have type one pili and flagella that can bind to host adhesion receptor carcinoembryonic antigen-related cell adhesion molecule 6 (CEACAM6) [74]. CEACAM6 has been shown to be overexpressed in ileal CD tissue compared to healthy controls, to be increased after IFN-*γ* or TNF-*α* stimulation, and to be upregulated by AIEC themselves [74]. AIEC have also been shown to possess long polar fimbriae and so can cross the mucosal barrier to access lymphoid cells [75]. AIEC LF82 bacteria isolated from an ileal CD patient exacerbate an inflammatory mucosal immune response involving upregulation of TLR5 (toll-like receptor) and IPAF flagellin receptors [76]. In CD patients, increased expression of CEACAM6 on the apical membrane of ileal enterocytes could promote the abnormal ileal mucosa colonization by AIEC bacteria, since CEACAM6 acts as a receptor for AIEC attachment to the intestinal mucosa [74]. In transgenic CEABAC10 mice expressing human CEACAMs to mimic the high expression of CEACAM6 reported in CD patients, the AIEC reference strain LF82 induced development of severe clinical symptoms of colitis in a type 1 pili dependent manner [77, 78]. Additionally, another AIEC strain NRG857c has been reported to colonize intestinal mucosa of conventional mice following streptomycin treatment, leading to chronic inflammation involving Th1 and Th17 responses and intestinal fibrosis [79]. The abnormal persistence of AIEC bacteria in this model could be related to their recently reported ability to actively resist antimicrobial peptides secreted by intestinal cells [80]. Of note, gut microbiota composition and host mucosal homeostasis are altered in CEABAC10 mice submitted to Western diet, favouring AIEC bacteria colonization of gut mucosa [81]. This is in line with the multifactorial etiology of CD and emphasizes the role of diet in CD pathogenesis.

### 2.4. Is AIEC an Instigator or a Propagator of Colitis?

**Figure 1:**
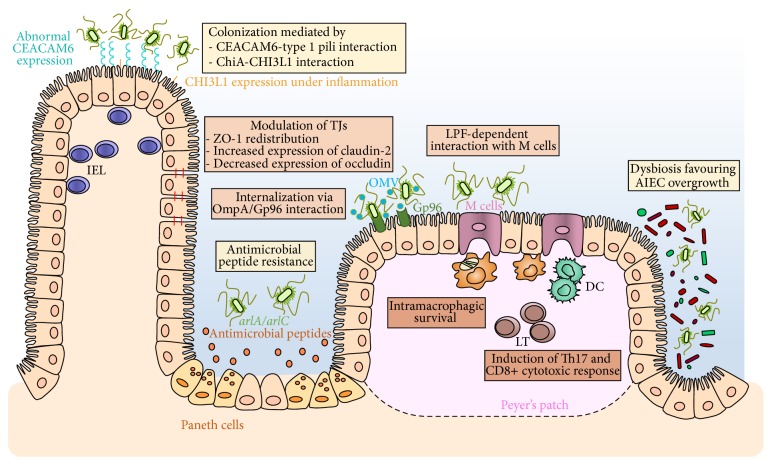
Strategies used by AIEC to trigger intestinal inflammation. (1) AIEC are able to strongly adhere to intestinal epithelial cells and colonize gut mucosa using type 1 pili that can bind to mannose residues of CEACAM6, which is overexpressed on the apical surface of ileal epithelial cells in patients with ileal CD. AIEC are also able to adhere to chitinase 3-like-1 receptor (CHI3L1)* via* the chitin-binding domain of ChiA bacterial protein. AIEC actively resist antimicrobial peptides secreted by Paneth cells. This mechanism involves two genes,* arlA*, which encodes a Mig-14 family protein implicated in defensin resistance, and* arlC*, an OmpT family outer membrane protease. (2) AIEC translocation through the epithelial barrier is increased following different mechanisms leading to exacerbation of intestinal inflammation. Modulation of tight junctions (TJs) by AIEC induces paracellular barrier permeability involving ZO-1 redistribution, increased expression of pore-forming claudin-2, and decreased expression of occludin. The endoplasmic reticulum (ER) stress response glycoprotein Gp96 is overexpressed at the apical membrane of ileal epithelial cells in CD patients and acts as a host-receptor for AIEC outer membrane vesicles (OMV) carrying OmpA protein promoting the invasion of the intestinal mucosa. AIEC bacteria interact with Peyer's patches and translocate across M cells* via* long polar fimbriae (LPF) expression to access lymphoid cells. (3) AIEC intramacrophagic replication is favored in the submucosal compartment of host cells. AIEC intramacrophagic survival could be due to host autophagy defects leading to increased bacterial replication and also enhancing inflammatory responses. AIEC can also induce Th17 and CD8+ cytotoxic responses.

At present, it is difficult to determine whether AIEC bacteria trigger intestinal inflammation, thus leading to the disease, or whether they colonize the gut mucosa as a consequence of preexisting inflammatory context in which case they could be an aggravating factor. AIEC colonized WT and TLR5KO mice only transiently but chronic colitis persisted in TLR5KO months later, suggesting that this microbe acted as an instigator, rather than a propagator, of colitis [82]. Inversely, inflammation leads to a shift from Gram+ to Gram−, a proliferation of mucosally invasive* E. coli* and a decrease in microbial diversity [83]. An answer concerning the origin of AIEC persistence may be proposed in the future with the development of fecal sample banks collecting specimens throughout life from patients who develop IBD and control subjects. With the current knowledge, AIEC bacteria are considered as an intestinal pathobiont able to promote disease only in specific host genetic or environmental contexts (Figure 1). Pathobionts were thus termed to distinguish them from acquired infectious agents [4, 84]. Host factors expressed specifically during intestinal inflammation have been shown to play major roles in facilitating infection with enteric bacteria and especially with AIEC. Ileal lesions in CD patients are colonized by pathogenic AIEC bacteria owing to the increased expression of a specific bacterial attachment molecule, CEACAM6, at the brush border of the ileal epithelium [78]. AIEC are also able to adhere to chitinase 3-like-1 receptor (CHI3L1)* via* the chitin-binding domain of ChiA bacterial protein promoting the pathogenic effect of AIEC in IBD [85]. AIEC outer membrane protein OmpA has been shown to interact with the endoplasmic reticulum (ER) stress response glycoprotein Gp96, which is also overexpressed at the apical membrane of ileal epithelial cells in CD patients [86]. Given that ER stress is commonly associated with inflammation [87], AIEC may also take advantage of the ER stress occurring in CD patients to increase adherence to the intestinal epithelium. AIEC delay apoptosis in infected macrophages, favoring their own persistence in CD patients, by a mechanism involving increase of S-nitrosylation and proteasomal degradation of caspase-3 [88]. In addition, AIEC bacteria modulate the ubiquitin proteasome system turnover in infected-intestinal epithelial cells by downregulating the NF-*κ*B regulator CYLD, leading to I*κ*B-*α* degradation and NF-*κ*B activation. This property plays a key role in the pathogenicity of AIEC since it favours intracellular replication of AIEC reference strain LF82 [89]. An abnormal autophagy, an innate defense mechanism allowing clearance of intracellular pathogens, could also favor AIEC persistence in the gut of IBD patients, as supported by several recent studies. Indeed, altered expression of ATG16L1, IRGM, or NOD2 favoured intramacrophagic replication of AIEC and led to enhanced secretion of IL-6 and TNF-*α* in response to AIEC infection [90]. Conversely, the numbers of intramacrophagic AIEC and proinflammatory cytokine release are strongly decreased upon pharmacological induction of autophagy [90]. Of note, autophagy is blocked at the autolysosomal step following AIEC infection of neutrophil-like PLB-985 cells, allowing intracellular survival of bacteria and increased IL-8 secretion [91]. Moreover, microRNAs MIR106B and MIR93 decrease the expression of ATG16L1 and prevent autophagy-mediated elimination of intracellular bacteria, a process that seems to be impaired in colonic mucosa of patients with active CD [92]. AIEC infection upregulated levels of microRNA- (MIR-) 30C and MIR130A in T84 cells and in mouse enterocytes, leading to reduced levels of ATG5 and ATG16L1 and to inhibition of autophagy, increased numbers of intracellular AIEC, and increased inflammatory response [66].

During the latter 20th century, increased CD incidence has been associated with consumption of polysaccharides in Western diets. AIEC LF82 specific biofilm formation was strongly favored in the presence of maltodextrin (MDX), a starch-derived polysaccharide [93]. MDX also promoted bacterial adhesion to human intestinal epithelial cells* via* a mechanism involving type 1 pili. However, this was independent of the expression of CEACAM6, indicating a distinct mechanism of AIEC adhesion to enterocytes [93]. As MDX is an ubiquitous dietary component, this suggests that Western diets, enriched in specific polysaccharides, may contribute to dysbiosis and lead to disease susceptibility. This is also supported by alteration of microbiota composition reported in CEABAC10 mice fed a Western diet [81].

The concept of pathobionts is supported by clinical data which reveal that, in IBD patients with underlying genetic mutations, inflammation may be driven by specific members of the microbiota rather than by infectious pathogens [94]. The analysis of AIEC genome revealed the presence of specific genes that could be involved in bacterial virulence, but pathoadaptive mutations in many other genes or bacterial DNA sequences could also participate in AIEC pathogenicity in a susceptible host [95, 96]. For example, OmpA proteins of LF82 bacteria interact with the host molecule Gp96 and allow adhesion to intestinal epithelial cells [86]. Recently acquired nonsynonymous substitutions are considered as a typical signature of the pathoadaptive evolution of bacterial pathogens and have notably been reported in FimH variants expressed by AIEC strains, conferring them higher adhesion ability [97]. FimH pathoadaptive mutations required for AIEC gut colonization have thus been selected, leading to the development of inflammation in a genetically susceptible host [97]. Therefore,* fimH* SNPs analysis could be a marker of virulence for IBD patients'* E. coli* strains and could be used for diagnosis or epidemiological studies. Moreover, new therapeutic strategies to impair AIEC adhesion to the gut mucosa in the early stages of IBD could be considered. Of interest, the protease meprin has been shown to degrade type 1 pili of AIEC bacteria and prevent their binding to mannosylated host-receptors [98]. Meprin expression is decreased in CD patients and this decrease correlates with the severity of inflammation, suggesting that the lack of protective meprin could favor AIEC colonization of gut mucosa [98]. AIEC, and perhaps other pathobionts, may therefore instigate chronic inflammation in susceptible hosts by altering the gut microbiota composition.

## 3. Crosstalk between Epithelial Barrier and Adherent-Invasive* E. coli*

The gastrointestinal epithelium forms a single-cell layer between the blood circulation and the external environment of the intestinal lumen [99]. Functions of intestinal barrier are to control uptake across the mucosa and to protect mucosa against intraluminal toxins, invading microorganisms, and lumen gut antigens [100]. The intestinal epithelium provides a physical barrier with interconnections between intestinal epithelial cells. The cell-cell contact is mediated by different protein complexes including adherens junctions, tight junctions, and desmosomes to stabilize the mechanical cohesion of the cells [101]. Also, epithelium acts as a chemical barrier through the intestinal mucus layer and the synthesis of antimicrobial peptides, an integral part of innate immunity. An efficient intestinal mucosal barrier is crucial for protection against the external environment. A barrier dysfunction has been characterized in patients suffering from IBD that leads to enhanced intestinal permeability [102]. Barrier disorders including defects in thickness or composition of the intestinal mucus layer, alterations of tight junctional complexes, and disturbances of the synthesis of antimicrobial peptides result in an inadequate protection of the epithelium against the adherence and invasion of luminal bacteria* via* specific receptors in the epithelium abnormally expressed in the context of intestinal inflammation [101]. Bacterial adhesion to and colonization of intestinal epithelial cells are considered as the crucial initializing steps in IBD pathogenesis before bacteria translocate and enter the submucosal compartment [85]. There is some evidence that, following invasion, AIEC bacteria can alter epithelial barrier function by displacing and redistributing ZO-1, a protein required for the formation of apical tight junctions [103]. The decrease in barrier integrity could result in an increase in AIEC translocation across the epithelial barrier leading to an exacerbation of AIEC pathogenesis [49]. Gut barrier damage and inflammatory responses are crucial for the perturbation and aggravation of intestinal inflammation [104].

### 3.1. Failure of the Intestinal Mucus Layer and Defective Production of Antimicrobial Peptides

The small intestine is lined with a thin mucus layer while the colon and stomach are covered by two layers of mucus [105]. The thinner inner layer (50–200 *μ*m) common to the stomach, small intestine, and colon is densely packed and strongly linked to the intestinal epithelium, which ensures its protection. This layer provides a matrix for the retention of antimicrobial peptides, including *α*-defensins, secreted by Paneth cells, thereby establishing a barrier between microorganisms and mucosal intestinal tissue. This inner layer prevents direct contact of the epithelium with the luminal microorganisms, whereas the outer layer, much thicker and difficult to dislodge, is colonized by a large number of commensal bacteria [105]. In IBD, deficiencies in mucus production and the secretion of antimicrobial peptides allow commensal bacteria to become opportunistic pathogens and contribute to chronic intestinal inflammation [106]. A reduced mucus layer has been reported to correlate with increased disease severity. Moreover, in IBD patients, the mucolytic species* Ruminococcus torques* and* Ruminococcus gnavus* have also been reported to be more prevalent and more abundant [107]. This disproportionate increase in mucolytic bacteria could explain the total increase in mucosa-associated bacteria in IBD since their ability to degrade human secretory mucin (MUC2) could promote the adhesion and invasion of opportunistic bacteria [107]. In CEABAC10 transgenic mice expressing human CEACAMs, Western diet led to a shift in microbiota composition comparable to what is observed in CD patients, with an increase in the mucin-degrading bacterium* Ruminococcus torques* and the* Bacteroides/Prevotella* group [81]. Western diet altered barrier function by decreasing* Mucin-2*,* Klf4,* and* Tff3* expression, mucus layer thickness, and goblet cell number in colonic mucosa. In these mice, AIEC bacteria have better ability to colonize the gut mucosa and to trigger intestinal inflammation due to alteration of barrier function and increased TNF-*α* secretion [81]. Moreover, some AIEC strains have been shown to resist antimicrobial peptides, which could promote their survival in the inner mucus layer [80]. A better understanding of the molecular mechanisms involved in increased intestinal permeability in CD patients with a Western diet, in the context of CD genetic susceptibility and in the presence of AIEC, should help the development of new drugs to target AIEC-induced disruption of intestinal barrier integrity.

### 3.2. Disruption of Epithelial Barrier Integrity by AIEC in the Context of CD

The intestinal epithelial cells are joined at their apical side by tight junctions (TJs), a space between the enterocytes that is finely regulated and is crucial in regulating intestinal permeability. Host-microbiota interactions promote a reorganization of TJs [108]. These interactions between pathogenic bacteria and epithelial tissues often disturb the intestinal TJs barrier and often lead to a number of pathophysiological disorders. The alteration of TJs protein content increases paracellular barrier permeability and contributes to intestinal inflammation. Recent studies showed a reduced number of tight junction strands and an increased number of strand breaks in CD patients [109]. Claudins are the major functional and structural components of TJs. Specifically, an increased expression of the pore-forming claudin-2 and a decreased expression of occludin were detectable [109, 110]. Moreover, in CEACAMs-expressing mice colonized with AIEC bacteria, an increase of intestinal permeability has been reported that could be related to the induction of claudin-2 expression consequently to AIEC/CEACAM6 interaction [110]. In a spontaneous model of IBD closely mimicking CD (SAMP1/YitFc mice), a dysregulation of the epithelial barrier function has also been shown, involving aberrant expression of claudin-2 and occludin and resulting in ileitis with a worsening of histological scores [111, 112]. Intestinal barrier function in CD patients may be restored by defending against type 1 pili-mediated AIEC/CEACAM6 interaction.

### 3.3. Peyer's Patches as Portals of Entry for AIEC Bacteria

Peyer's patches (PPs) play a major role in mucosal immunity. CD pathogenesis results from an inadequate innate and/or adaptative immune response to the microflora supported by the relationship between PPs and CD lesions [113]. PPs have a defined role in the interaction between immune response and microbiota and consequently participate in intestinal epithelial disorders [113]. Their interplay with the diversity and the function of the gut microbiota is becoming an effective area of research [113]. A number of pathogenic microorganisms have evolved original strategies to pass through the apical epithelial barrier and penetrate into the intestinal epithelium. Many studies now indicate that several microorganisms, particularly invasive pathogens, use specialized M cells as the primary portal of entry into the host to cross the intestinal barrier and initiate the disease. For example,* Yersinia enterocolitica* and* Yersinia pseudotuberculosis* cross the intestinal epithelial barrier by adhering to M cells of the follicle-associated epithelium [114].* Salmonella* Typhimurium also invade M cells and thus access to the PPs, although they have also been reported to be sampled by dendritic cells extending transcellular protrusions through M cells [115, 116]. PPs were suspected to be the site of initial inflammation and thus to play a major role in the early stages of CD disease [117]. A recent study showed that AIEC interact with PPs and translocate through M cells* via* long polar fimbriae (LPF); moreover, the prevalence of LPF-expressing AIEC strains was higher among CD patients than among control subjects [75]. Following translocation through the epithelium, AIEC bacteria could be internalized into immune cells, especially macrophages and dendritic cells, which are able to release inflammatory mediators such as TNF-*α* that can drive functional alterations of the mucosal barrier and lead to general mucosal permeability defects [100].

## 4. Handling of Microbiota-Derived Antigens in Health and Inflammatory Bowel Diseases

The intestinal mucosa contains high numbers of effector lymphocytes, including IgA-producing plasma cells and effector CD4+ T cells, among them IFN-*γ*-producing Th1 cells, IL-17-producing Th17 cells, and Foxp3-expressing regulatory T cells (Tregs), but also CD8+ T cells and intraepithelial lymphocytes (mainly *γ* *δ* T cells) [118]. In addition, recent studies have revealed the importance of innate lymphoid cells that share functional characteristics with T cells [119].

### 4.1. Microbial Containment by Mucosal Firewall

The intestinal immune system needs to ensure simultaneously the immune tolerance of microbiota and host defense against microbial invasion whether by pathogens or by commensals taking advantage of occasional barrier weakness. Among other mechanisms, this is achieved by the reciprocal regulation of inflammatory and regulatory immune responses [120]. Inflammatory responses are kept under control by FoxP3+ Tregs originating either from the thymus or from local differentiation of naive CD4+ T cells. Surprisingly, a recent report suggested that intestinal Tregs are mainly of thymic origin and not locally induced following exposure to commensal or diet antigens as previously assumed [121]. Of note, innate lymphoid cells have also been shown to play a major role in the regulation of effector T cell responses to commensals [122]. Thus, at steady-state, the “mucosal firewall,” comprising mucus layer, epithelial barrier, IgA, and regulatory cells, contains microbiota- and food-derived antigens and limits inappropriate immune responses [123]. Microbiota-derived antigens do not, therefore, normally stimulate systemic immunity [124]. Captured either directly or more probably transferred from other mucosal phagocytes, these antigens are then handled by CD103+ dendritic cells, known to preferentially induce Treg differentiation and homing to the gut mucosa through the production of retinoic acid [125]. Pathogens are then recognized on the basis of their invasiveness and sensed by pattern-recognition receptors (PRR) expressed either in an intracellular way or in the basolateral compartment of the epithelium [126]. Any disruption of the mucosal firewall, even local and transient, caused, for instance, by acute infection, epithelial damage, or just by increased permeability, could thus allow for microbial translocation and impair discrimination between commensals and pathogens. In line with this, it is known that acute infection can lead to loss of tolerance to commensals and induction of microbiota-specific T cells with inflammatory phenotype [127]. Immune memory cells are probably generated following this kind of event and could be reactivated, as suggested by the detection of antibodies to microbiota in healthy human serum [128]. How the mucosal firewall is restored following such events and whether commensal-specific memory T cells remain and take part in the characteristic alternation of clinical relapse and remission in IBD still need to be elucidated.

### 4.2. Reciprocal Shaping of Microbiota and Mucosal Immunity

Commensal flora plays an essential part in the development of gut-associated lymphoid structures, that is, PPs and isolated lymphoid follicles, as shown by studies on germ-free animals [129]. Microbiota has also been recognized as a major regulator of mucosal immune system activation and tuning, through direct interactions with epithelial or immune cells or by producing immunomodulatory metabolites [120].

The microbiota stimulates regulatory responses, mainly through the production of active metabolites.* Clostridium* clusters IV and XIVa, which are decreased in IBD patients' flora, are known to induce colonic IL-10-producing cells [130, 131]. Belonging to this group of microorganisms,* Faecalibacterium prausnitzii* secrete a still unidentified factor exerting anti-inflammatory effects* in vitro* on human intestinal epithelial cells and peripheral blood mononuclear cells [20]. In addition, intragastric administration of* F. prausnitzii* or their supernatant ameliorates TNBS colitis in mice [20]. An increase in Treg frequency was also reported recently in mice fed probiotics such as* Lactobacillus reuteri* [132]. Bacterial fermentation products, namely, short chain fatty acids (SCFA) such as butyrate and acetate produced by Bacteroidetes phylum or Clostridia, suppress inflammation and have a protective effect against colitis by favoring the differentiation and function of colonic Tregs in a GPR43-dependent manner [133–135]. Other bacterial products such as polysaccharide A from nonenterotoxinogenic* Bacteroides fragilis* also promote expansion of IL-10-producing FoxP3+ Tregs and ameliorate colitis in mice [136, 137].

Conversely, evidence of effector T cell stimulation by microbiota has also been documented. Segmented filamentous bacteria (SFB) closely adhere to PPs and potently stimulate Th17 differentiation in mice, even though they also induce other types of helper T cells [138, 139]. Other microbial products such as bacterial DNA also stimulate Th17 activation at steady-state [140]. These ROR*γ*T+, IL-17 secreting CD4+ T cells are often considered detrimental for the host owing to their recurrent implication in inflammatory and autoimmune pathogenesis. For instance, Th17 response to enterotoxinogenic* Bacteroides fragilis* induces colitis and tumor formation in Min mice and SFB colonization has also been reported to aggravate autoimmune arthritis in mice [141, 142]. However, severe DSS-induced colitis in ROR*γ*T deficient mice is reversed by antibiotic treatment suggesting a crucial role for Th17 cells in microbiota containment and mucosal homeostasis at steady-state [143]. In addition, Th17 response clearly plays a protective role against fungal and bacterial enteric pathogens such as* Citrobacter rodentium* [138]. Thus, Th17 responses probably contribute to intestinal homeostasis at steady-state and become pathogenic only in particular contexts such as a host autoimmune susceptibility or immune overstimulation due to massive bacterial translocation. According to this view, the increased expression of IL-17 and IL-22 reported in CD patients' mucosa would not necessarily mean a pathogenic role for these cytokines but could rather be the signature of an abnormal stimulation of Th17 antimicrobial immunity. The deregulated expansion of Th17 population, combined with the reinforcement of their proinflammatory role by IL-23, would make them become harmful and participate in the development of inflammatory diseases [144].

Thus, gut microbiota seems to play a dual role in immune regulation. By stimulating the adaptive immune system and promoting the generation of different T cell subsets in the gut mucosa, normal intestinal flora contributes to immune homeostasis while being detrimental in autoimmune models such as arthritis and experimental autoimmune encephalomyelitis [136, 141]. Of note, Treg cells in gut mucosa are specific to microbiota-derived antigens and any perturbation of microbiota composition strongly influences Treg repertoire [121]. Likewise, antibody repertoire has been shown to be progressively shaped by changes in microbiota and to adjust to the latest microorganisms present [145]. Reciprocally, the shaping of microbiota composition by the immune system has been demonstrated in several mouse models, among them NOD2-deficient mice, PGRP- (peptidoglycan recognition protein-) deficient mice, and TRUC mice, in which immune defects induce the establishment of a colitogenic flora able to transfer colitis to healthy mice [146–148].

### 4.3. Antigenic Stimulation of Adaptive Immunity in Inflammatory Bowel Diseases

IBD is generally considered as the result of an inappropriate response of the adaptive immune system to microbiota-derived antigens. This view is supported by the fact that most genetic polymorphisms associated with a higher risk of IBD affect the responsiveness of the mucosal immune system to microorganisms [149]. This excessive stimulation is thought to induce effector T cell responses: CD has long been considered as a Th1 disease with increased levels of TNF-*α*, IFN-*γ*, and IL-12 in patients' inflamed mucosa whereas increased concentrations of Th2 cytokines such as IL-5 and IL-13 are rather a feature of UC [150–152]. This notion was next challenged by the recognition of excessive Th17 infiltration and elevated concentrations of IL-23, IL-17, IL-22, and IL-21 in patients' inflamed mucosa. A gain-of-function mutation on IL23R gene has been reported to predispose patients to both CD and UC whereas IL-17 secretion by PBMC of patients correlates with disease severity in UC but not in CD, suggesting a major role of the IL-23 in the pathogenesis of IBD but possibly a different involvement of Th17 cells in the two disorders [153, 154]. Of note, IL-23 not only sustains Th17 response but also promotes Il-17 and IFN-*γ* secretion by innate lymphoid cells, leading to colitis in mice [155]. These innate lymphoid cells are abnormally represented in IBD subjects mucosa compared to healthy controls [156]. A functional plasticity between Th1 and Th17 lineage has also been proposed and is consistent with the identification in CD patients of pathogenic Th1/Th17 cells releasing both IL-17 and IFN-*γ* [157]. Thus, Th1 and Th2 responses are currently considered as the true immunopathogenic components driving inflammation, respectively, in CD and UC, whereas Th17 response could initiate the deregulation of these effector responses [152].

While the exact nature of antigens stimulating the immune system remains elusive, evidence for microbiota-induced activation of B cell and T cell immunity does not suggest the involvement of a unique, pathogenic antigen in disease etiology but rather a generalized loss of tolerance to microbiota in IBD subjects. Elevated levels of antibodies directed against microbial structures, among them antibodies to* Saccharomyces cerevisiae* (ASCA) and to* E. coli* membrane protein OmpC and flagellin, have been found in IBD patients and associated with aggressive forms of the disease [158]. These systemic antibodies have also been detected in patients' unaffected relatives, in whom they are predictive of IBD development [159]. Of note, although there is no data available concerning the possible correlation between AIEC colonization and OmpC antibodies in CD patients, both have been specifically associated with ileal involvement [44, 160]. However, even if their concentration is strongly increased in IBD, antibodies against gut-resident flora also occur in healthy subjects, probably following transient barrier dysfunction [128]. Whether these responses are cross-reactive antibodies against conserved bacterial antigens is currently not known. Nevertheless the simple presence of microbiota-specific systemic immunity clearly cannot account by itself for the development of IBD.

Regarding T cell response and in line with what has been found for antibody response, the diversity of the TCR repertoire is affected in CD patients and oligoclonal expansions of CD4+ T cells persisting after surgery have been detected in inflamed as well as noninflamed mucosa [161]. No common TCR specificity has been identified among patients, suggesting there is no shared antigenic response at the origin of these abnormal T cell oligoclonal proliferations. Of note, chronic infection with adherent-invasive* E. coli* has been reported to induce Th17 and cytotoxic T cell responses in mice [79]. Altogether, this favors a decisive role of a pathogenic T cell response in the triggering of inflammation in gut mucosa and in postoperative recurrence.

Current knowledge is in line with a multiple-hit model in which IBD triggering is due to neither host susceptibility nor the environment nor the microbiota but due to the concomitant occurrence of intestinal barrier dysfunction that allows abnormal antigenic stimulation of immunity in a susceptible host prone to mount uncontrolled inflammatory responses.

## 5. New Treatment Options for CD Patients: Beyond Manipulation of Immune Response, Targeting of AIEC and Restoration of Gut Microbiota?

Current CD treatments including immunosuppressive agents and synthetic anti-TNF-*α* or anti-integrin antibodies mainly focus on reducing the symptoms but are unable to treat the cause of the disease insofar as the origin of initial inflammation remains elusive. Despite recent advances in this field, the available drugs are not devoid of side effects and a large subset of CD patients do not respond or undergo loss of responsiveness in the course of their disease [162]. Beyond improvement of diarrhea and abdominal pain, new therapeutic goals now intend to limit mucosal damage and promote mucosal healing in order to achieve long-term deep remission. In the past decade, many promising advances have been made and extensively reviewed in the field of IBD [162–164].

### 5.1. Immunomodulation Treatments

Besides the major breakthrough of the discovery of TNF-*α* antagonists, the blockade of inflammatory cytokines or their receptors did not reach expectations, with the exception of some therapeutic antibodies targeting the p40 subunit of IL-12 and IL-23, IL-6 receptor, or IL-13, whose efficacy still has to be confirmed [164]. Likewise, systemic administration of anti-inflammatory cytokines such as IL-10 did not result in clinical improvement [165]. Numerous other possible therapeutic agents have been suggested for IBD treatment, including agonists of peroxisome proliferator-activated receptor gamma (PPAR*γ*) modulators and elafin, an endogenous regulator of protease activity, thereby underlining an urgent need for efficient and safe drug-delivery systems to the gut mucosa [166, 167]. Although this approach does not appear feasible with current regulations, oral administration of genetically modified food-grade bacteria has been considered for local expression of therapeutic molecules at the mucosal surface [168, 169].

New strategies are currently emerging to dampen or manipulate the abnormal immune response in IBD patients. The manipulation of the T cell costimulatory pathway by anti-CD28 antibody Abatacept did not work [170]. However, the humanized antibody to *α*4*β*7 integrin Vedolizumab, which prevents homing of immune cells to gut mucosa, has yielded encouraging results in CD [171]. Among innovative strategies in this field, the injection of activated regulatory T cells into CD patients has shown promising potential and could open up the way to personalized immunotherapy [172]. Of note, autologous hematopoietic stem cell transplantation has also been reported to induce durable remission in CD patients refractory to conventional therapies [173–175].

Therapeutic interventions targeting dysbiosis in general and/or AIEC colonization in particular are promising for changing the natural CD history. Today the manipulation of patient microbiota through antibiotherapy, fecal transplantation, nutritional interventions, or pre/probiotic administration could be used either alone or in combination with immunotherapy to induce remission in active disease or as a postoperative therapy to prevent relapse.

### 5.2. Antibiotics

The use of antibiotics is currently restricted to bacterial complications of CD because their efficacy has not been clearly established and because of their side effects [176]. However, antibiotics may induce remission in active CD, especially in patients with colonic involvement [177, 178]. Their efficacy in preventing postoperative recurrence has also been studied, with conflicting results [179, 180]. Antibiotics nevertheless deserve further evaluation in IBD, notably in particular cases such as CD patients with evidence of AIEC colonization. Additionally, in the future it may become possible to specifically target antibiotic activity against aggressive bacterial species, thus reducing side effects and allowing selective elimination of undesirable bacteria.

### 5.3. Fecal Transplantation

Fecal microbiota transplantation is another drastic way of modifying the patient's microbiota and has been successfully tested in recurrent* Clostridium difficile* infections [181, 182]. By restoring essential components of intestinal flora, it could reverse the inappropriate immune stimulation in CD and make intestinal ecosystem less suitable for AIEC intestinal colonization. The mode of delivery and the preparation of donor stools still have to be perfected, and the potential long-term consequences of fecal transplantation remain to be established. Its safety and efficacy in CD patients are currently under investigation, especially in those for whom standard treatments have failed [183]. Nevertheless, the efficacy of fecal transplantation has recently been attested to in CD patients in independent studies [184, 185].

### 5.4. Probiotics, Prebiotics, and Postbiotics

As a promising way of modulating microbiota composition, the administration of presumed anti-inflammatory probiotics has been tested in CD [186]. Their ability to induce remission has not been clearly demonstrated but they may be effective in maintaining remission in postoperative prophylaxis [187–189]. The use of a yeast probiotic has recently been reported to prevent colitis in mice and therefore could represent a new strategy to treat patients with ileal CD that are abnormally colonized by AIEC [190]. As a complementary strategy, nondigestible prebiotics could also be given to stimulate growth or metabolic activity of beneficial microbial species; this approach is currently under investigation in CD patients [191]. New experimental models consisting of polarized explants of healthy or IBD gut mucosa have recently been developed to assess* ex vivo* the effect of probiotics [192]. In these models, some probiotics worsened inflammation in IBD mucosal explants, probably because of increased permeability and higher bacterial translocation, which suggests that the administration of probiotics to IBD patients should be considered with great caution [192]. The safety of probiotic use in active CD has also been recently questioned by the report of a break of tolerance to commensal flora in acute inflammatory context [127]. As an alternative to probiotics, the use of postbiotics (soluble factors produced by probiotics and able to elicit immunomodulatory response) as therapeutic agents could be of interest in CD since they could be administered in a purified and well-characterized form to guarantee their safety [186]. There is thus a need to identify bacterial immunomodulatory factors like, for instance, lactocepin, a serine protease secreted by* Lactobacillus casei* that decreases inflammation in a murine colitis model through selective degradation of proinflammatory cytokines, and to deliver them to the intestinal mucosa in a safe form [193].

### 5.5. Phage Therapy

Finally, the abundance and diversity of bacteriophage communities in the human gut have recently been investigated and certain differences between the bacteriophage colonization of IBD patients and that of healthy controls strongly suggest a possible implication of bacteriophages in IBD [194]. New animal models have been developed to study the dynamics of phage/bacterial communities in the gut that open up a new area of research and therapeutic possibilities [195].

## 6. Conclusion

Despite original therapeutic options available, current CD treatments have important limitations with regard to safety, efficacy, and applicability and often cause severe side effects. Anti-TNF-*α* have been involved in fatal blood disorders, infections, and liver injury. In the near future, a better understanding of microbiota function in intestinal inflammation will provide new therapeutic opportunities to treat CD patients. There is an accumulation of evidence that CD probably occurs as a result of inappropriate triggering of the mucosal immune system in a host genetic and/or epigenetic susceptibility and under certain dietary or environmental conditions. The potential multiplicity of etiologies suggests that CD patients need personalized therapeutic strategies: a subgroup of CD patients abnormally colonized by AIEC could benefit from specific treatment aiming at eradicating these bacteria. More generally, there is an urgent need to identify biomarkers that can reliably predict the responsiveness and efficacy of treatments. Microbial signatures could prove to be useful as such biomarkers for diagnosis, for monitoring disease activity, and for therapeutic orientation [196].

## Figures and Tables

**Table 1:** Abnormal prevalence of *Escherichia coli* in Crohn's disease patients.

Date	Country	Method	Sample	References
1978	United Kingdom	Antibody	Blood	[35]
1978	United Kingdom	Culture	Ileal and colonic biopsies	[36]
1995	United States of America	Immunocytochemical	Intestines and mesenteric lymph node specimens	[37]
1997	The Netherlands	DNA probe	Rectal biopsies and feces	[38]
1998	France	Culture	Ileal biopsies	[39]
2001	France	Ribotyping	Ileal biopsies	[40]
2002	Japan	qPCR^1^	Small and large intestine and ileocolitis biopsies	[41]
2004	Ireland	Nested PCR	Microdissected granulomas	[42]
2004	United Kingdom	Culture	Ileal, ileocolonic, and colonic biopsies	[43]
2004	France	Culture	Ileal biopsies	[44]
2005	United Kingdom	FISH^2^	Rectal biopsies	[45]
2006	United States of America	OmpC Antibody	Serum	[46]
2007	Canada	Culture	Ileocolonic and colonic biopsies	[47]
2007	United Kingdom	qPCR^1^	Ileal biopsies	[48]
2007	United States of America	Culture	Colonic biopsies	[49]
2009	France	qPCR^1^	Ileal biopsies	[50]
2009	Sweden	qPCR^1^	Ileal biopsies	[51]
2009	Denmark	Culture	Feces	[52]
2010	Germany	Cloning	Colonic biopsies	[53]
2010	Germany	qPCR^1^	Feces	[54]
2010	Australia	Microarray	Feces	[55]
2011	France	qPCR^1^	Feces	[56]
2011	Brazil	Culture	Rectal biopsies	[57]
2012	Brazil	Culture	Ileal, colonic, and rectal biopsies	[58]
2013	United States of America	Culture	Ileal biopsies	[59]
2013	China	qPCR^1^	Feces	[60]
2013	United Kingdom	culture	Ileal, ileocolonic, and colonic biopsies	[61]
2014	Spain	qPCR^1^	Ileal, ileocolonic, and colonic biopsies	[23]
2014	Australia	qPCR^1^	Ileal biopsies	[62]

^1^Quantitative polymerase chain reaction; ^2^fluorescent in situ hybridization.
